# Supplementary material for: Modeling glioblastoma heterogeneity as a dynamic network of cell states
Source: Mol Syst Biol. 2021 Sep 16;17(9):e10105. doi: 10.15252/msb.202010105 (PMC8444284; doi:10.15252/msb.202010105)
Supplement: Supplementary file 5 — Source Data for Figure 3 [file MSB-17-e10105-s001.zip › Figure3A_sourcedata/GSEA_3065/nosigdb_state4.GseaPreranked.1622718430994/GAROFANO_IAVARONE_MTC_SUBTYPE.html]

Details for gene set GAROFANO\_IAVARONE\_MTC\_SUBTYPE[GSEA]

|  || Dataset | state4 |
| Phenotype | NoPhenotypeAvailable |
| Upregulated in class | na\_pos |
| GeneSet | GAROFANO\_IAVARONE\_MTC\_SUBTYPE |
| Enrichment Score (ES) | 0.450801 |
| Normalized Enrichment Score (NES) | 2.046641 |
| Nominal p-value | 0.0 |
| FDR q-value | 8.073015E-4 |
| FWER p-Value | 0.005 |
Table: GSEA Results Summary

  

Fig 1: Enrichment plot: GAROFANO\_IAVARONE\_MTC\_SUBTYPE      
 Profile of the Running ES Score & Positions of GeneSet Members on the Rank Ordered List

  

| PROBE | GENE SYMBOL | GENE\_TITLE | RANK IN GENE LIST | RANK METRIC SCORE | RUNNING ES | CORE ENRICHMENT || 1 | NLRP1 |  |  | 16 | 0.425 | 0.0305 | Yes |
| 2 | TOMM7 |  |  | 22 | 0.380 | 0.0593 | Yes |
| 3 | IRS2 |  |  | 33 | 0.338 | 0.0841 | Yes |
| 4 | TSC22D4 |  |  | 49 | 0.298 | 0.1048 | Yes |
| 5 | PHPT1 |  |  | 63 | 0.282 | 0.1247 | Yes |
| 6 | PFDN5 |  |  | 78 | 0.265 | 0.1431 | Yes |
| 7 | CLU |  |  | 95 | 0.252 | 0.1601 | Yes |
| 8 | UQCRB |  |  | 114 | 0.241 | 0.1760 | Yes |
| 9 | RPS27L |  |  | 149 | 0.223 | 0.1878 | Yes |
| 10 | BLOC1S1 |  |  | 170 | 0.214 | 0.2012 | Yes |
| 11 | COX6B1 |  |  | 185 | 0.209 | 0.2152 | Yes |
| 12 | HIGD2A |  |  | 207 | 0.202 | 0.2275 | Yes |
| 13 | HIGD1A |  |  | 216 | 0.198 | 0.2416 | Yes |
| 14 | BPGM |  |  | 223 | 0.193 | 0.2557 | Yes |
| 15 | RAB9A |  |  | 254 | 0.185 | 0.2652 | Yes |
| 16 | CDKN1A |  |  | 319 | 0.171 | 0.2681 | Yes |
| 17 | RPS15 |  |  | 320 | 0.171 | 0.2814 | Yes |
| 18 | CMBL |  |  | 345 | 0.166 | 0.2904 | Yes |
| 19 | PPP2R2B |  |  | 399 | 0.152 | 0.2936 | Yes |
| 20 | RPL41 |  |  | 411 | 0.150 | 0.3034 | Yes |
| 21 | MAPK8IP1 |  |  | 433 | 0.146 | 0.3114 | Yes |
| 22 | H3F3B |  |  | 447 | 0.144 | 0.3205 | Yes |
| 23 | SEMA6D |  |  | 454 | 0.143 | 0.3307 | Yes |
| 24 | SRI |  |  | 458 | 0.143 | 0.3413 | Yes |
| 25 | JUND |  |  | 513 | 0.135 | 0.3429 | Yes |
| 26 | PJA2 |  |  | 563 | 0.129 | 0.3450 | Yes |
| 27 | ALDH3A2 |  |  | 578 | 0.128 | 0.3527 | Yes |
| 28 | MLC1 |  |  | 594 | 0.126 | 0.3601 | Yes |
| 29 | PLTP |  |  | 625 | 0.123 | 0.3647 | Yes |
| 30 | KIF21A |  |  | 627 | 0.123 | 0.3741 | Yes |
| 31 | POLR2I |  |  | 658 | 0.119 | 0.3785 | Yes |
| 32 | CHCHD5 |  |  | 679 | 0.117 | 0.3844 | Yes |
| 33 | BTG2 |  |  | 689 | 0.116 | 0.3919 | Yes |
| 34 | HEPACAM |  |  | 703 | 0.113 | 0.3986 | Yes |
| 35 | ACYP2 |  |  | 708 | 0.113 | 0.4068 | Yes |
| 36 | SYT17 |  |  | 716 | 0.112 | 0.4143 | Yes |
| 37 | BBOX1 |  |  | 823 | 0.102 | 0.4049 | Yes |
| 38 | PFKM |  |  | 825 | 0.102 | 0.4127 | Yes |
| 39 | AMZ2 |  |  | 868 | 0.098 | 0.4135 | Yes |
| 40 | NDUFS7 |  |  | 887 | 0.097 | 0.4181 | Yes |
| 41 | SOX21 |  |  | 930 | 0.093 | 0.4185 | Yes |
| 42 | GAMT |  |  | 971 | 0.090 | 0.4189 | Yes |
| 43 | BTBD2 |  |  | 991 | 0.089 | 0.4227 | Yes |
| 44 | CINP |  |  | 1003 | 0.088 | 0.4278 | Yes |
| 45 | CA2 |  |  | 1037 | 0.085 | 0.4291 | Yes |
| 46 | FRS2 |  |  | 1058 | 0.084 | 0.4323 | Yes |
| 47 | DTNA |  |  | 1063 | 0.084 | 0.4382 | Yes |
| 48 | TIMM13 |  |  | 1143 | 0.079 | 0.4314 | Yes |
| 49 | NDUFS4 |  |  | 1144 | 0.079 | 0.4376 | Yes |
| 50 | SLC4A4 |  |  | 1160 | 0.078 | 0.4412 | Yes |
| 51 | SH3PXD2B |  |  | 1181 | 0.077 | 0.4440 | Yes |
| 52 | TRIAP1 |  |  | 1240 | 0.074 | 0.4402 | Yes |
| 53 | DHRSX |  |  | 1254 | 0.073 | 0.4438 | Yes |
| 54 | PRR5L |  |  | 1260 | 0.073 | 0.4487 | Yes |
| 55 | MRPL54 |  |  | 1282 | 0.072 | 0.4508 | Yes |
| 56 | CRLS1 |  |  | 1322 | 0.070 | 0.4498 | No |
| 57 | FZD3 |  |  | 1429 | 0.065 | 0.4376 | No |
| 58 | NUDT16L1 |  |  | 1471 | 0.063 | 0.4357 | No |
| 59 | TSPAN3 |  |  | 1584 | 0.059 | 0.4220 | No |
| 60 | UBE2F |  |  | 1606 | 0.058 | 0.4231 | No |
| 61 | ENOPH1 |  |  | 1638 | 0.057 | 0.4225 | No |
| 62 | PRCP |  |  | 1675 | 0.056 | 0.4209 | No |
| 63 | RNF141 |  |  | 1697 | 0.055 | 0.4218 | No |
| 64 | DCTN4 |  |  | 1702 | 0.055 | 0.4255 | No |
| 65 | VPS37D |  |  | 1747 | 0.054 | 0.4224 | No |
| 66 | CKB |  |  | 1774 | 0.053 | 0.4223 | No |
| 67 | NME5 |  |  | 1778 | 0.053 | 0.4259 | No |
| 68 | ALDH6A1 |  |  | 1803 | 0.052 | 0.4261 | No |
| 69 | ZNF24 |  |  | 1805 | 0.052 | 0.4300 | No |
| 70 | ELOF1 |  |  | 1846 | 0.051 | 0.4274 | No |
| 71 | THAP2 |  |  | 1878 | 0.050 | 0.4262 | No |
| 72 | MAP2K5 |  |  | 1880 | 0.050 | 0.4299 | No |
| 73 | STK33 |  |  | 1886 | 0.050 | 0.4330 | No |
| 74 | DDR1 |  |  | 1941 | 0.048 | 0.4279 | No |
| 75 | ZNF626 |  |  | 1950 | 0.048 | 0.4304 | No |
| 76 | HINT3 |  |  | 2016 | 0.047 | 0.4234 | No |
| 77 | NIPAL3 |  |  | 2069 | 0.045 | 0.4183 | No |
| 78 | DIRAS1 |  |  | 2072 | 0.045 | 0.4215 | No |
| 79 | UQCRFS1 |  |  | 2090 | 0.044 | 0.4222 | No |
| 80 | MOAP1 |  |  | 2096 | 0.044 | 0.4248 | No |
| 81 | PREX1 |  |  | 2110 | 0.044 | 0.4260 | No |
| 82 | PTPRA |  |  | 2113 | 0.043 | 0.4291 | No |
| 83 | VPS16 |  |  | 2117 | 0.043 | 0.4320 | No |
| 84 | GAN |  |  | 2173 | 0.042 | 0.4263 | No |
| 85 | STAU2 |  |  | 2181 | 0.042 | 0.4284 | No |
| 86 | C12orf73 |  |  | 2197 | 0.042 | 0.4292 | No |
| 87 | ZNF561 |  |  | 2233 | 0.041 | 0.4266 | No |
| 88 | RCHY1 |  |  | 2267 | 0.040 | 0.4243 | No |
| 89 | PCCB |  |  | 2285 | 0.040 | 0.4246 | No |
| 90 | ALDH5A1 |  |  | 2286 | 0.040 | 0.4277 | No |
| 91 | CCND2 |  |  | 2367 | 0.038 | 0.4175 | No |
| 92 | ATP6V0E2 |  |  | 2393 | 0.037 | 0.4163 | No |
| 93 | ECSIT |  |  | 2394 | 0.037 | 0.4192 | No |
| 94 | KCTD6 |  |  | 2407 | 0.037 | 0.4201 | No |
| 95 | PLEKHF2 |  |  | 2426 | 0.036 | 0.4200 | No |
| 96 | ISCA2 |  |  | 2477 | 0.035 | 0.4146 | No |
| 97 | RAB39B |  |  | 2503 | 0.035 | 0.4132 | No |
| 98 | C1orf53 |  |  | 2507 | 0.035 | 0.4154 | No |
| 99 | TFPT |  |  | 2583 | 0.033 | 0.4057 | No |
| 100 | TIMM9 |  |  | 2592 | 0.033 | 0.4069 | No |
| 101 | NDFIP1 |  |  | 2664 | 0.031 | 0.3978 | No |
| 102 | TMEM147 |  |  | 2715 | 0.030 | 0.3919 | No |
| 103 | ZNF181 |  |  | 2743 | 0.030 | 0.3898 | No |
| 104 | SLC27A5 |  |  | 2796 | 0.029 | 0.3835 | No |
| 105 | NUDT16 |  |  | 2831 | 0.028 | 0.3801 | No |
| 106 | CMTM4 |  |  | 2854 | 0.027 | 0.3786 | No |
| 107 | TRIM23 |  |  | 2885 | 0.027 | 0.3758 | No |
| 108 | ZNF84 |  |  | 2893 | 0.027 | 0.3768 | No |
| 109 | TRIM4 |  |  | 2971 | 0.025 | 0.3661 | No |
| 110 | DIS3L |  |  | 3011 | 0.025 | 0.3616 | No |
| 111 | ELMO2 |  |  | 3058 | 0.024 | 0.3560 | No |
| 112 | FBXL17 |  |  | 3080 | 0.023 | 0.3544 | No |
| 113 | G6PC3 |  |  | 3156 | 0.022 | 0.3438 | No |
| 114 | ABHD6 |  |  | 3158 | 0.022 | 0.3453 | No |
| 115 | ZNF677 |  |  | 3166 | 0.022 | 0.3459 | No |
| 116 | C18orf32 |  |  | 3206 | 0.021 | 0.3411 | No |
| 117 | PTPMT1 |  |  | 3216 | 0.021 | 0.3413 | No |
| 118 | TMEM199 |  |  | 3239 | 0.020 | 0.3392 | No |
| 119 | LFNG |  |  | 3245 | 0.020 | 0.3399 | No |
| 120 | MED9 |  |  | 3297 | 0.018 | 0.3330 | No |
| 121 | HKR1 |  |  | 3330 | 0.017 | 0.3291 | No |
| 122 | ZNF426 |  |  | 3336 | 0.017 | 0.3297 | No |
| 123 | ZNF134 |  |  | 3373 | 0.016 | 0.3250 | No |
| 124 | RASA4 |  |  | 3374 | 0.016 | 0.3263 | No |
| 125 | CAMK2D |  |  | 3403 | 0.016 | 0.3229 | No |
| 126 | MRPS26 |  |  | 3457 | 0.014 | 0.3153 | No |
| 127 | FARS2 |  |  | 3514 | 0.012 | 0.3070 | No |
| 128 | BAIAP2 |  |  | 3566 | 0.010 | 0.2994 | No |
| 129 | ZNF432 |  |  | 3570 | 0.010 | 0.2997 | No |
| 130 | DNAJC18 |  |  | 3572 | 0.010 | 0.3003 | No |
| 131 | PCIF1 |  |  | 3583 | 0.009 | 0.2994 | No |
| 132 | ZNF790 |  |  | 3605 | 0.009 | 0.2966 | No |
| 133 | TMEM59L |  |  | 3617 | 0.008 | 0.2955 | No |
| 134 | TMEM205 |  |  | 3636 | 0.008 | 0.2931 | No |
| 135 | OSBPL6 |  |  | 3642 | 0.007 | 0.2929 | No |
| 136 | NDUFS3 |  |  | 3667 | 0.006 | 0.2894 | No |
| 137 | TMEM131 |  |  | 3697 | 0.005 | 0.2851 | No |
| 138 | TMEM106B |  |  | 3704 | 0.005 | 0.2845 | No |
| 139 | MTHFS |  |  | 3717 | 0.004 | 0.2829 | No |
| 140 | CYP46A1 |  |  | 3740 | 0.004 | 0.2796 | No |
| 141 | UPF1 |  |  | 3773 | 0.002 | 0.2745 | No |
| 142 | ZNF350 |  |  | 3837 | -0.001 | 0.2642 | No |
| 143 | ASCC3 |  |  | 3973 | -0.008 | 0.2427 | No |
| 144 | ZNF606 |  |  | 4022 | -0.011 | 0.2356 | No |
| 145 | CDS2 |  |  | 4066 | -0.013 | 0.2296 | No |
| 146 | CSNK2A1 |  |  | 4151 | -0.019 | 0.2173 | No |
| 147 | PGGT1B |  |  | 4206 | -0.022 | 0.2102 | No |
| 148 | SLC12A4 |  |  | 4280 | -0.027 | 0.2003 | No |
| 149 | ZNF721 |  |  | 4449 | -0.040 | 0.1759 | No |
| 150 | STMN3 |  |  | 4493 | -0.044 | 0.1723 | No |
| 151 | JAG1 |  |  | 4496 | -0.044 | 0.1754 | No |
| 152 | NUDT9 |  |  | 4655 | -0.056 | 0.1539 | No |
| 153 | FJX1 |  |  | 4678 | -0.058 | 0.1548 | No |
| 154 | CYCS |  |  | 4894 | -0.075 | 0.1253 | No |
| 155 | ZYX |  |  | 4967 | -0.080 | 0.1198 | No |
| 156 | C19orf25 |  |  | 5169 | -0.099 | 0.0945 | No |
| 157 | TMEM68 |  |  | 5288 | -0.109 | 0.0837 | No |
| 158 | EGFR |  |  | 5316 | -0.110 | 0.0878 | No |
| 159 | REEP5 |  |  | 5378 | -0.116 | 0.0869 | No |
| 160 | TMED1 |  |  | 5380 | -0.116 | 0.0958 | No |
| 161 | AIG1 |  |  | 5419 | -0.121 | 0.0990 | No |
| 162 | RAD51C |  |  | 5525 | -0.134 | 0.0923 | No |
| 163 | DDRGK1 |  |  | 5640 | -0.151 | 0.0854 | No |
| 164 | PLD5 |  |  | 5924 | -0.213 | 0.0556 | No |
Table: GSEA details [plain text format]

  

Fig 2: GAROFANO\_IAVARONE\_MTC\_SUBTYPE: Random ES distribution      
 Gene set null distribution of ES for **GAROFANO\_IAVARONE\_MTC\_SUBTYPE**
